# Data-Driven Dental, Oral, and Craniofacial Analytics: Here to Stay

**DOI:** 10.1177/00220345221120564

**Published:** 2022-09-26

**Authors:** F. Schwendicke, M.L. Marazita

**Affiliations:** 1 Department of Oral Diagnostics, Digital Health, Health Services Research, Charité – Universitätsmedizin, Berlin, Germany; 2 Center for Craniofacial and Dental Genetics, Department of Oral and Craniofacial Sciences, School of Dental Medicine, and Department of Human Genetics, School of Public Health, University of Pittsburgh, Pittsburgh, PA, USA

**Keywords:** artificial intelligence, big data, bioinformatics, biostatistics, deep learning/machine learning, data science

Dental, oral, and craniofacial (DOC) health care and research are generating an increasing amount of data that are expected to be used to foster a deeper understanding of our patients’ health and disease, thus allowing more effective, efficient, and safer care. Some even postulate that data and data analytics may shape something coined “data dentistry,” with data-driven approaches and applications disseminating widely and deeply into DOC research and practice (Schwendicke and Krois 2021). Data are generated not only prospectively (e.g., in scientific studies)—with such prospectively collected data being increasingly large (e.g., omics or imagery data sets)—but also during clinical routine, involving history data, clinical records, images, specific tests, imaging, prescriptions, and so forth.

Big Data and data analytics in all its forms, including artificial intelligence (AI), and machine learning, have been successfully used in medicine and dentistry in recent years. The *Journal of Dental Research* has received an ever-increasing number of submissions involving such analytical strategies both for research and for clinical applications. This special issue of the *Journal of Dental Research* focuses on such aspects of DOC Big Data and advanced data analytics, aiming to display not only the breadth of data and analytical strategies currently used in DOC, their translational efforts, and their promised impact but also the challenges and current difficulties in the field as applied to DOC.

The explosion of truly Big Data in biology and health care began with the Human Genome Project (https://www.genome.gov/human-genome-project), an international initiative spearheaded by public-private research partnerships, with the goal of mapping the entire human genome (Watson 1990; Gibbs 2020). The project was envisioned in the 1980s, took off in the 1990s (Watson 1990), was considered finished in the early 2000s despite many gaps remaining (Lander et al. 2001), with a final complete assembly published in 2022 (Nurk et al. 2022). The data and tools developed from the Human Genome Project are publicly available, notably through the National Centerfor Biotechnology Information (https://www.ncbi.nih.gov/genome/guide/human/). The Human Genome Project remains the largest collaborative biological project in human history; it has spawned countless offshoots and has made possible today’s initiatives in precision health care and genomic research, including in DOC (Marazita 2012).

To fulfill national and international goals in precision health care, there have been concomitant data collection effortsover many years to collect and make available data from population- or cohort-based electronic health records, coupled with genomic, metabolomic, and many more “omics” to allow extremely large-scale projects investigating the biological and epidemiological underpinnings of human health and disease conditions. A relatively early example of such resources is the UK Biobank established in the early 2000s (www.ukbiobank.ac.uk), which contains accessible in-depth genetic and health information—including dental (Galloway 2011)—from half a million UK participants. More recently, the US has launched the All of Us project (allofus.nih.gov), with the goal of enrolling 1 million participants with electronic health records, imaging, and whole-genome sequencing. To date, All of Us has enrolled about 330,000 participants. Many similar projects are ongoing around the world, and 1 article in this special issue (Divaris et al. 2022) includes a description of a consortium of such projects focused on DOC conditions. Similarly, other authors of articles in this special issue summarize existing and available large biological data resources, for example, the online data repository FaceBase (facebase.org; Schuler et al. 2022) and the developing Human Oral and Craniofacial Cell Atlas (Caetano et al. 2022), and a new tool to visualize the gene and miRNA expression patterns across developmental stages developed from CleftGenDB data as well as regulatory networks (Yan et al. 2022).

Using these large, structured or unstructured data sets of different modalities, ideally also recorded repeatedly over time, is challenging, but undoubtedly worthwhile. Traditional analytic tools may not fully leverage the wealth of information in these data sets or may not even be able to work with such Big Data at all. To use such data fully and appropriately, there has been a concomitant explosion in data analytics, notably including the application of AI. This special issue highlights the application of AI for developing clinically relevant tools, for example, evaluating factors contributing to the long-term survival of root canal treatments using data from the National Dental Practice Based Research Network (Thyvalikakath et al. 2022), the diagnosis of oral squamous cell carcinoma (Yanget al. 2022), automated 3-dimensional (3D) cephalometric landmarking (Dot et al. 2022), the prediction of 3D postorthodontia facial changes in adults (Park et al. 2022), the predictive classification of tooth removal procedures (de Graaf et al. 2022), and the association of components of computer-aided design/computer-aided manufacturing resins with clinical outcomes (Li et al. 2022).

In addition to the development of clinical tools and assessments, other articles in this special issue address the application of analytical strategies to Big Data for research purposes in DOC science, for example, evaluating the impact of early childhood oral health on later academic performance (Wehby 2022); B/plasma cell infiltration in periodontitis, which may lead to a molecular diagnosis (Huang et al. 2022); novel AI network analyses to identify multimorbidity clusters in people with periodontitis (Larvin et al. 2022); and the value of information and economics around AI for caries detection (Schwendicke, Cejudo Grano de Oro, et al. 2022).

Harnessing Big Data presents challenges beyond analytic pathways and methods: data may not be accessible at all or, they are if accessible, may not conform to current FAIR (Findable, Accessible, Interoperable, and Reusable) principles, often being siloed due to data protection reasons or not usable given limited interoperability. Peer-reviewing research using such data, analyzed using the described complex (and often not fully explainable) methods, has been found to be challenging and may require new strategies for researchers and publishers alike (Schwendicke, Marazita, et al. 2022). Efforts toward accessing DOC data or using them, even if they are not directly accessible, are required, in concert with harmonizing data formats and analytical streams. The authors in this special issue address a variety of such issues, for example, difficulties in appropriately benchmarking different AI applications (Schneider et al. 2022), the lack of interoperability of available DOC data (Rajkumar et al. 2022), DOC research data availability and quality according to the FAIR principles (Uribe et al. 2022), harmonizing caries disease phenotypes for large-scale genomics studies (Divaris et al. 2022), federated learning in dentistry (Rischke et al. 2022), conceptualization of social epigenomics for oral health (Gomaa 2022), and trustworthy AI in DOC research (Ma et al. 2022).

DOC is uniquely positioned to embrace advanced data analytics, with a wealth of routinely collected, longitudinal data being complemented by a wide range of large prospectively collected data sets to allow deeper insights into individuals’ health. This special issue displays the breadth of research in our domain along with its chances, achievements, and challenges. The *Journal of Dental Research* remains focused on fostering innovative, data-driven science in the field and to help unlock the potential of Big Data and advanced analytics for driving better DOC health care for all.

## Author Contributions

F. Schwendicke, M.L. Marazita, contributed to conception, design, data acquisition, analysis, and interpretation, drafted and critically revised the manuscript. All authors gave final approval and agree to be accountable for all aspects of the work.
